# Flat-Band Potential Determination and Catalytical Properties of Sn_3_O_4_/SnO_2_ Heterostructures in the Photo-Electrooxidation of Small Organic Molecules under Ultraviolet (370 nm) and Blue (450 nm) Light

**DOI:** 10.3390/ma16237300

**Published:** 2023-11-23

**Authors:** Evgeny Gribov, Evgeny Koshevoy, Aleksey Kuznetsov, Maxim Mikhnenko, Evgeniy Losev, Mikhail Lyulyukin

**Affiliations:** 1 Boreskov Institute of Catalysis SB RAS, Pr. Lavrentieva 5, 630090 Novosibirsk, Russia; koshevoy@catalysis.ru (E.K.); kan@catalysis.ru (A.K.); m.mikhnenko@catalysis.ru (M.M.); losev.88@mail.ru (E.L.); lyulyukin@catalysis.ru (M.L.); 2 Faculty of Natural Sciences, Chair of Solid-State Chemistry, Novosibirsk State University, Pirogova Str. 1, 630090 Novosibirsk, Russia; 3 Voevodsky Institute of Chemical Kinetics and Combustion SB RAS, Laboratory of Nanoparticles, Institutskaya Str. 3, 630090 Novosibirsk, Russia

**Keywords:** Sn_3_O_4_, Sn_3_O_4_/SnO_2_, band gap, photo-electrooxidation, acetone, glycerol, methanol

## Abstract

Sn_3_O_4_ are promising semiconductor materials due to their visible light absorption ability. In this work, a series of materials, such as SnO_2_, Sn_3_O_4_ and Sn_3_O_4_/SnO_2_ heterostructures, with different phase ratios were prepared using hydrothermal synthesis. The materials were characterized using X-ray diffraction (XRD), Raman and diffuse reflectance spectroscopy (DRS), high resolution transmission electron microscopy (HRTEM), nitrogen adsorption (BET). Flat-band potentials (E_FB_) of the samples were determined using the photocurrent onset potential (POP) method. It was shown that the potentials obtained with open circuit potential measurements versus illumination intensity (OCP) likely corresponded to the E_FB_ of SnO_2_ nanoparticles in heterostructures due to interfacial electron transfer from the conducting band of Sn_3_O_4_ to that of SnO_2_. The photo-electrooxidation processes of a series of organic substrates were studied in the potential range of 0.6–1.4 V vs. RHE under irradiation with ultraviolet (λ = 370 nm) and visible (λ = 450 nm) light. The Sn_3_O_4_ sample showed high activity in the photo-electrooxidation of acetone and formic acid in visible light. The Sn_3_O_4_/SnO_2_ samples exhibited noticeable activity only in the oxidation of formic acid. The presence of the SnO_2_ phase in the Sn_3_O_4_/SnO_2_ samples increased the photocurrent values under ultraviolet illumination, but significantly reduced the oxidation efficiency in visible light.

## 1. Introduction

Tin dioxide is a promising material with high corrosion stability, high oxidation potential, low toxicity and high economic efficiency [1,2]. SnO_2_ is widely used as supports for fuel cell catalysts [3], sensors [4], electrodes for supercapacitors [5], conductive transparent material [6], photocatalysts and electrocatalysts for the oxidation of organic substances [1,2,7]. However, the large band-gap energy, about 3.6 ÷ 4 eV, and the recombination of photogenerated electrons and holes [8] do not allow the material to be widely used for working in either visible or solar light and lead to low values of quantum efficiency. Approaches to broadening the wavelength range of light absorption and reducing recombination process comprise doping with various metals and nonmetals, including tin itself, mixed valency tin oxide (e.g., Sn_3_O_4_), heterojunction engineering, solid solution with another oxide materials and morphology control [2,9]. With these approaches, an increase in efficiency is achieved either by introducing new energy levels into the band gap or by reducing the band-gap width, which leads to a shift in the absorption edge to the visible region [2].

Sn_3_O_4_ and composites based on it are promising materials that arouse increased interest in various photo-stimulated and electrochemical processes, including hydrogen production [10,11,12,13,14], water decomposition [15], oxidation of dyes and organic compounds [16,17,18,19,20,21], sensors [22,23,24], lithium and sodium ion batteries [25,26,27,28], supercapacitors [29], solar cells [30], CO_2_ reduction [31,32], visible light photodetectors [33], etc. The morphology of Sn_3_O_4_ represents a layered structure in which two layers of SnO alternate with a layer of SnO_2_ [34]. The presence of Sn^2+^ ions leads to the appearance of absorption in the visible-light region [34] and also forms a morphology as nanosheets [12] or nanowires [13], which significantly increase the specific surface area and its availability for the reaction. The ability to vary the band gap and the position of the conduction band and, hence, the oxidation potential associated with the position of the valence band by adding hydrogen peroxide during synthesis has been shown theoretically and confirmed experimentally [12]. Despite this, the main disadvantage for the use of Sn_3_O_4_ is that the potential of the valence band is not high enough for photo-oxidation reactions. To increase the efficiency of charge separation, composites of Sn_3_O_4_ with electrically conductive materials based on graphene [10,35], graphene oxide [14,32], Ni foam [19], Sn [20] and heterostructures based on C_3_N_4_ [17], SnO [36,37], SnO_2_ [22,24,38,39,40] and Si nanowires [41] are being studied intensively. Heterostructures based on high valence band oxidation potential of SnO_2_ with an absorbance in the visible region of Sn_3_O_4_ can increase the efficiency of photocatalytic reactions. SnO_2_-Sn_3_O_4_ heterostructures were used as sensors and showed high sensitivity to NO_2_ due to a large number of oxygen vacancies and improved electron transfer [22]. The structures of Sn_3_O_4_/SnO_2_ were studied in the reaction of CO_2_ electroreduction to formic acid. The increased efficiency is attributed to the increased adsorption energy of intermediates at the SnO_2_ and Sn_3_O_4_ interface [39]. SnO_2_–Sn_3_O_4_ heterostructures were used as sensors for formaldehyde. The improved performance was attributed to the high concentration of adsorbed oxygen involved in oxidation and the characteristics of the heterophase Schottky junction [24]. SnO/Sn_3_O_4_ and SnO_2_/Sn_3_O_4_ heterostructures were studied in the photooxidation of rhodamine B. The authors concluded that such heterostructures are promising in photocatalysis due to the presence of heterojunctions [36].

Despite the many works on Sn_3_O_4_, the processes of photo-electrooxidation of organic substances on such materials have been practically not considered. Photoelectrochemical studies are mainly devoted to the characterization of materials with photocurrent values [14,42] and the determination of the position of the flat-band potential with the Mott–Schottky method [13,14,16,32,33,41]. The photo-electrooxidation of rhodamine B on vertically aligned Sn_3_O_4_ nanoflakes grown on carbon paper was studied under visible light irradiation. The authors associated the increase in photocurrent with the presence of channels for electron transport and the accessibility of the surface [33]. Sn_3_O_4_ was studied in the hydrogen evolution reaction. It was discovered that polarization of the electrode and treatment of the material with phosphoric acid leads to an increase in the efficiency of charge separation and a decrease in photo-corrosion of the material due to a difficulty in capturing holes from Sn (II) ions [43].

In this work, a series of Sn_3_O_4_/SnO_2_ samples with varying phase contents was synthesized. The conduction band potentials were measured using a set of photoelectrochemical methods, and the obtained data were compared. The photo-electrocatalytic properties of materials were studied in the oxidation of a number of simple organic substances (methanol, acetone, formic acid, glycerol) at different potentials under ultraviolet and visible light.

## 2. Materials and Methods

### 2.1. Materials

The following materials were used in the work: Sodium citrate pentahydrate (99.99%, special purity, LLC “Khimkraft”), SnCl_2_*2H_2_O (analytical grade, JSC “Vekton”), NaOH (special purity, LLC “Component-Reaktiv”), ethanol (J. T. Baker, >99.9% reagent grade), HCl (special purity 35–38 wt.%, Sigma-Tek LLC), acetone (EKOS-1, special purity grade), formic acid (Sigma-Aldridge, reagent grade >95%), glycerol (analytical grade, JSC Vekton), methanol (J. T. Baker, (Ultra) Gradient HPLC grade), Nafion (5 wt.% solution in ethanol, Sigma-Aldrigh).

### 2.2. Synthesis

Samples TO1 and TO2 were synthesized in accordance with ref. [20]. An amount of 25 mmol of sodium citrate and 10 mmol of tin (II) chloride were dissolved in 50 mL of water with stirring, and 25 mL of 0.2 M sodium hydroxide solution was slowly added. Stirring of the solution continued for 20 min (sample TO1). For sample TO2, the same procedure was followed without the addition of sodium citrate. For sample TO3, the synthesis procedure was slightly modified in accordance with ref. [10]. An amount of 0.01 mol of tin (II) chloride were dissolved in 75 mL of water with stirring, and 25 mL of 4 M sodium hydroxide solution was slowly added. The solution was stirred continuously for 20 min. Then, 6.5 mL of concentrated hydrochloric acid was slowly added to the resulting clear solution. The resulting solutions were transferred to a Teflon autoclave liner (150 mL) and placed in an oven. Hydrothermal synthesis was carried out at 180 °C for 12 h.

For comparison, SnO_2_ was synthesized (sample TO4). An amount of 3.960 mL of 50 wt.% NaOH solution was diluted to 71.040 mL with water, followed by adding 1.7028 g of tin (II) chloride under vigorous stirring. The resulting clear solution was transferred to a Teflon autoclave liner (150 mL) and placed in an oven. Hydrothermal synthesis was carried out at 150 °C for 24 h.

The resulting yellow-brown (TO1 and TO3), dirty yellow (TO2) and white (TO4) precipitates were separated from the solution and washed 2 times with 0.2 M sodium hydroxide solution and 2 times with ethanol using a centrifuge. The powders were dried in an oven at 60 °C for 12 h.

### 2.3. Physico-Chemical Characterization

The porous structure was analyzed with low-temperature nitrogen adsorption at 77 K on an ASAP 2400 specific surface area analyzer (Micromeritics, Norcross, GA, USA). X-ray diffraction patterns were obtained using a Thermo ARL X’tra diffractometer (Thermo Fisher Scientific Inc., Ecublens, Switzerland) using a Mythen2R 1D linear detector (Dectris AG, Baden-Daettwil, Switzerland) with CuK_α_ radiation (λ = 1.5418 Å). The value of the average coherent scattering region (CSR) was calculated as the average value over two peaks at 24 and 37° 2θ using the Scherrer formula D = λ/(βcos(θ)), where β is the peak width at half maximum, without taking into account instrumental broadening. The lattice parameters were refined using the POLYCRYSTAL software package [44]. XPS spectra were recorded on a SPECS X-ray photoelectron spectrometer (SPECS Surface Nano Analysis GmbH, Berlin, Germany) using non-monochromatic MgK_α_ radiation (hν = 1253.6 eV). UV–Vis diffuse reflectance spectra (DRS) were recorded using a Cary 300 UV–Vis spectrophotometer (Agilent Technologies Inc., Santa Clara, CA, USA). Raman spectra were recorded using a Horiba Jobin Yvon LabRAM HR spectrometer coupled with an Olympus BX41 optical microscope, an argon laser (wavelength 488 nm) and a CCD Symphony detector (Horiba Ltd., Kyoto, Japan) in backscatter geometry. The HRTEM study was carried out using ThemisZ electron microscope (Thermo Fisher Scientific, Waltham, MA, USA).

### 2.4. Photoelectrochemical Studies

Catalyst suspension was obtained using ultrasonication of 10 mg of sample and 0.1 mg of Nafion (as ethanol solution) in 0.6 mL of deionized water. Then, suspensions were supported on FTO with drop-casting followed by drying and thermal treatment at 100 °C for 30 min to obtain a uniform layer with mass ratio of ~1.5 mg cm^–2^ and good stability in Na_2_SO_4_ electrolyte. Before the measurements, the sample was mounted to the cell and irradiated in air for 30 min with UV light to completely oxidize the remaining organic impurities. Before each experiment, the potential of mercury sulfate electrode was measured relative to reversible hydrogen electrode (RHE), and the potentials presented in the work were recalculated relative to RHE.

Experiments were performed in homemade three-electrode cell using potentiostat Autolab PG302N with a frequency response analyzer. Mercury sulfate electrode, Pt foil and fluorinated tin oxide (FTO) glass coated with photocatalyst were applied as reference, counter and working electrodes, respectively. An amount of 1 M Na_2_SO_4_ and 1 M Na_2_SO_4_ + 1 M EtOH were used as electrolytes. Ethanol as hole trap was added in order to reduce recombination effect. High power (100 W) light emitting diodes (LEDs) with a maximum irradiation at 370 nm and 450 nm were used as light sources.

Photo-electrocatalytic oxidation of a series of organic substrates (formic acid, methanol, glycerol and acetone) with concentration of 0.1 M was carried out at constant photon flux of 2 × 10^16^ cm^−2^ s^−1^ in the potential range of 0.6–1.4 V vs. RHE by alternating light phase (30 s)–dark phase (30 s). Impedance measurements were recorded at different potentials using frequency range of 1–50,000 Hz with 10 mV of voltage amplitude. Capacity was calculated according to Equation (1):

(1) $$C=\frac{1}{2\pi fZ^{\prime \prime }},$$

where C was capacity (F/g), f was frequency (Hz), Z′′ was imaginary part of impedance (Ohm).

Flat-band potential values (E_FB_) were obtained with three independent Mott–Schottky (MS) plot, open-circuit-potential (OCP) and photocurrent-onset-potential (POP) methods. The values obtained will be referred to as E_MS_, E_OCP_ and E_POP_, respectively. Since these values obtained are quite different, as will be shown later, the correct value of flat-band potential proposed in the work will be denoted as E_FB_.

E_MS_ was determined using Equation (2):

(2) $$C_{sc}^{-2}=\frac{2}{N_{d}e_{0}\varepsilon_{0}\varepsilon_{s}S^{2}}\left(E-E_{MS}-\frac{kT}{e_{0}}\right),$$

where C_sc_ (F/g) was the depletion layer capacity, N_d_ (m^−3^) was the charge carriers density, *e*_0_ was the electron charge (1.6 × 10^–19^ C), ε_0_ was the vacuum permittivity (8.85 × 10^–12^ F m^–1^), ε_s_ was the dielectric constant of material, S (m^2^) was the electrode surface area, E (V) was the potential of electrode, k (1.381·× 10^–23^ J K^–1^) was the Boltzmann constant, T (K) was the temperature.

E_POP_ was determined from chopped illumination (3 s) of the catalysts at 370 nm when recording cyclic voltammograms with scan rate of 1 mV/s. E_POP_ was obtained at potential when positive and negative spikes of photocurrents become equal. E_OCP_ was determined from the dependence of OCP of catalyst irradiated by UV light (λ = 370 nm) on light intensity, which ranged from 3 to 190 mW/cm^2^. E_OCP_ was estimated by extrapolation of measured potentials data to infinite light power density.

## 3. Results

### 3.1. Physico-Chemical Properties

**Figure 1:**
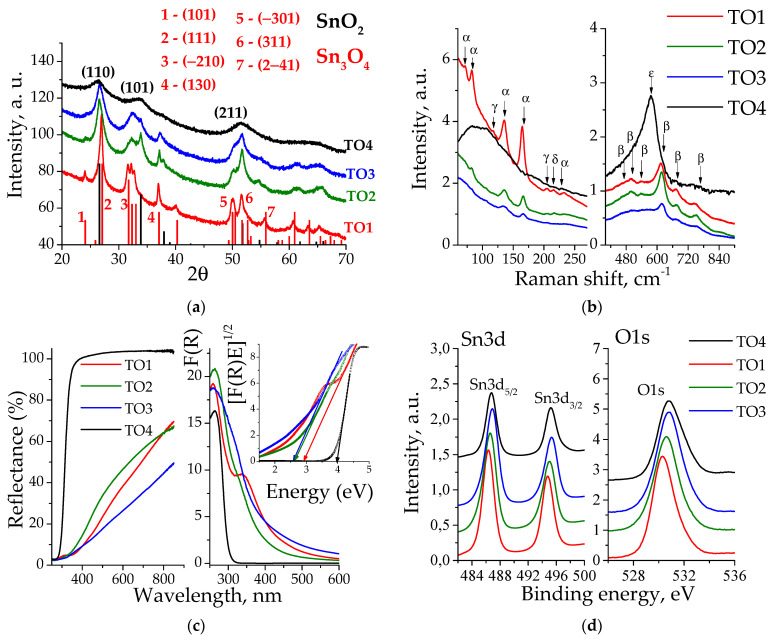
(**a**) XRD patterns. Bars show reflections and relative intensities of SnO_2_ (black) and Sn_3_O_4_ (red) phases. Indexes of main reflections are also presented; (**b**) Raman shifts in the wavenumber range of 60–270 cm^−1^ (**left**) and 400–900 cm^−1^ (**right**). Greek letters indicate phases found: α—Sn_3_O_4_, β—SnO_2_, γ—SnO, δ—Sn_3_^II^O_2_(OH)_2_ [45], ε—SnO_2_; (**c**) Spectra of DRS (**left**), Kubelka–Munk transformed (**right**) and Tauc plots (inset); (**d**) XPS spectra in Sn3d (**left**) and O1s (**right**) regions.

The XRD analysis (Figure 1a) showed that TO1 represents the triclinic phase Sn_3_O_4_ (P_−1_, PDF #16-0737), while TO4 only showed the SnO_2_ phase (P42/mnm, PDF #41-1445). Samples TO2 and TO3 contain SnO_2_ and Sn_3_O_4_ phases in different proportions. Table S1 shows the calculated cell parameters. For sample TO1, all observed peaks are slightly shifted to smaller angles relative to those of the Sn_3_O_4_ phase, which indicates an increase in unit cell parameters due to the possible presence of water molecules. For samples TO2 and TO3, the observed positions coincide quite well with the literature’s data. The coherent scattering region (CSR) values and phase composition estimates are presented in Table 2 and discussed below.

The Raman spectra (Figure 1b) of a SnO_2_ phase are characterized by the presence of main peaks at 631 cm^−1^ (A_1g_), 773 cm^−1^ (B_2g_) and 474 cm^−1^ (E_g_). [46,47,48]. For sample TO4, a large band is observed at 576 cm^−1^ (ε), characteristic of an amorphous phase with small particle sizes and surface defects [49,50,51]. The presence of 3 nm nanoparticles in TO4 sample is confirmed with XRD data (Table 2). A large number of β peaks with low intensity corresponds to surface phonon vibrations [47]. TO2 and TO3 samples exhibit α peaks at 135 cm^−1^ and 167 cm^−1^. For the pure Sn_3_O_4_ phase (sample TO1), the intensity of these peaks increases, and new ones appear at 72 cm^−1^, 83 cm^−1^. All these peaks are assigned to the Sn_3_O_4_ phase [16,19,20,23]. Of the three low intensity peaks observed at 205 cm^−1^, 215 cm^−1^ and 233 cm^−1^, the last one belongs to the Sn_3_O_4_ (α) phase. A similar peak at 241 cm^−1^ was observed for Sn_3_O_4_ in refs. [23,25]. The peak at 215 cm^−1^ (δ) may arise due to the presence of hydroxyl groups. A similar band was reported at 224 cm^−1^ for Sn_3_^II^O_2_(OH)_2_ in ref. [45]. Impurities of the SnO phase (γ) were noted at 118 cm^−1^ and 205 cm^−1^ [48]. Since the SnO phases for all samples and the SnO_2_ phase for TO1 were not observed in the XRD spectrum, it can be assumed that these species may be present in small quantities in the form of amorphous impurities.

In the DRS spectra (Figure 1c), the presence of the Sn_3_O_4_ phase results in the visible-light absorption appearance. In Kubelka–Munk coordinates, the spectra are characterized by a wide absorption tail in the visible region extended to more than 600 nm (less than 2 eV). The complex structure of the spectra in Tauc coordinates is most likely caused by the complex structure of energy levels in the band gap due to the interaction of the SnO_2_ and SnO layers. From the Tauc equation, the band-gap values for indirect transitions were determined in accordance with the literature’s data [15,27,52]. The results are present in Table 2. The XPS spectra are shown in Figure 1d, while the decomposition into spectral components are shown in Figure S1. The TO4 sample in the Sn3d region is characterized by the presence of a doublet at 486.8 eV and at 495.2 eV, which correspond to Sn3d_5/2_ and Sn3d_3/2_ bands, respectively. Decomposition of the band at 486.8 eV shows only the Sn^4+^ form of ions, of which the energy, according to the literature’s data, lies at 486.9 eV [53]. In the oxygen region, decomposition produces bands at 530.7, 532.2 and 535.5 eV associated with lattice oxygen [53,54,55], surface OH groups and/or chemisorbed oxygen [56,57] and with adsorbed water, respectively. The Sn3d_5/2_ bands of other samples consist of two components at 486.6–486.9 eV and 485.9–486.1 eV, corresponding to Sn^4+^ and Sn^2+^ ions, respectively [25,26]. The O1s band decomposes into two components at 530.2 eV (O1) and at 531.5 eV (O2). The assignment of bands of oxygen groups is quite difficult. In some studies, the O1 and O2 bands are assigned to oxygen groups associated with Sn^2+^ and Sn^4+^, respectively [21,28]. Since in this work the intensity of the O1 and O2 bands does not correlate with the content of these ions, we attribute O1 to lattice oxygen groups and O2 to chemisorbed oxygen in accordance with ref. [24,25]. The calculated surface composition of the samples is presented in Table 1.

**Figure 2:**
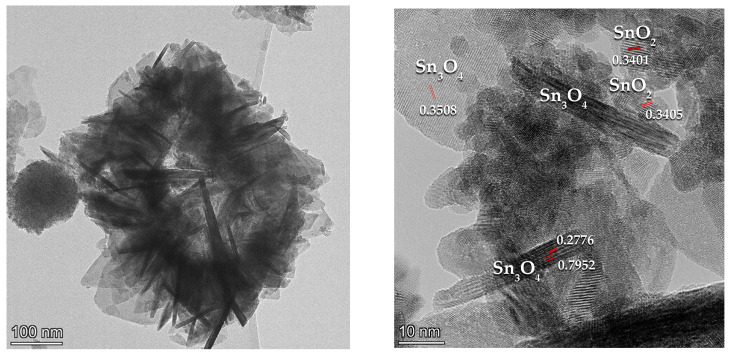
High-resolution TEM microphotographs of TO3 sample in low (**left**) and high (**right**) resolution.

According to Table 1, the surface chemical composition of the TO1 sample is Sn_3_O_3.75_, which is close to the ideal Sn_3_O_4_. A higher Sn^4+^ content (46%) than expected (33%) indicates the presence of small amounts of amorphous SnO_2_ on the surface, which is confirmed by Raman spectra. For samples TO2 and TO3, an increase in the amount of Sn^4+^ is observed, which correlates with the XRD data, where the content of the SnO_2_ phase increases. Sample TO4 has the formula SnO_1.88_ and corresponds to the SnO_2_ phase. The small size of TO4 particles (CSR 3 nm, Table 2) results in a high content of defective surface oxygen groups. Figure 2 shows the HRTEM data for the TO3 sample. Differently oriented Sn_3_O_4_ nanosheets with dimensions of the order of several tens of nanometers and a thickness of about 5 nm are well observed.

The SnO_2_ phase consists of nanoparticles of 5–10 nm in size, which are in close contact with the surface of Sn_3_O_4_ nanosheets. The found nanoparticle sizes correlate with the CSR sizes obtained for this sample with XRD (Table 2). Table 2 summarizes the data obtained with the methods discussed above.

TO1 and TO4 samples represent Sn_3_O_4_ and SnO_2_ phases with CSR sizes of 27 and 3 nm, respectively. The band-gap values obtained are consistent with the literature for these phases: 2.94 eV for Sn_3_O_4_ [10] and 3.98 eV for SnO_2_ [58]. The specific surface area of the TO4 sample is very low (2.05 m^2^/g), which could be associated with the rigid structure of small-sized SnO_2_ particles (3 nm). It is noteworthy that the incorporation of SnO_2_ enhances the specific surface area of Sn_3_O_4_/SnO_2_ heterostructures, as reflected by an increase in the specific surface area from 38.5 m^2^/g to 95.9 m^2^/g with an increase in the SnO_2_ phase content from 0% to 62%.

### 3.2. Flat-Band Potential Determination

**Figure 3:**
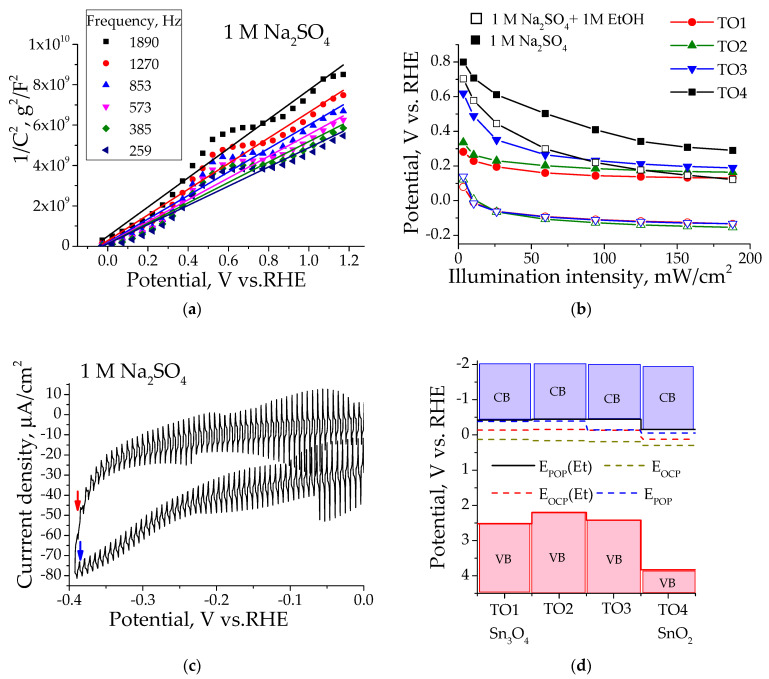
(**a**) MS plot obtained for TO1 sample; (**b**) Dependence of the open-circuit potential on the light intensity (OCP method) in pure electrolyte (full symbols) and with the addition of ethanol (empty symbols). The errors in determining potentials are ± 15 mV for all samples; (**c**) POP method for TO1 sample in pure electrolyte; (**d**) Potential values of the conduction band (CB), calculated valence band (VB) and potential levels, obtained by POP and OCP methods in pure electrolyte and with the addition of ethanol (Et).

The Mott–Schottky (MS), photocurrent onset (POP) and open-circuit potential (OCP) methods were used to determine flat-band potentials in pure electrolyte and with ethanol additives. The obtained data are presented in Figures S2 and S3 and in Figure 3 for TO1 sample. The potentials values obtained are compared in Table 3.

For the MS method, several frequencies were used, and the resulting E_MS_ values were averaged. The observed differences in potential values for different methods may be due to various reasons. E_MS_ values were found to differ significantly from those of other methods. Apparently, the MS method is not applicable in our case for various reasons, which may include the influence of particle size, the influence of the FTO substrate, the presence of two phases with different characteristics and morphology and others [59,60,61]. In the OCP method, as was shown earlier [60], the determined potential can be limited by defect levels below the conduction band, at which rapid recombination occurs. We previously suggested that, for TiO_2_-N samples, methanol additions reduce the degree of recombination due to an interaction with holes, and the detected E_OCP_ becomes close to the E_POP_ corresponding to E_FB_ [60]. Previously, we also showed that the POP method for monophase samples can be recommended as the principal one for measuring E_FB_ [60]. Based on these considerations, we assume that the E_FB_ values for the studied samples correspond to the E_POP_ values measured in the presence of the sacrificial agent ethanol. The obtained E_FB_ values for samples containing the Sn_3_O_4_ phase showed similar values of about −0.43 V and did not depend on the SnO_2_ content. For pure SnO_2_ (TO4), the E_FB_ value was found to be −0.15 V.

In the literature, E_FB_ values were estimated either from data on the position of the Sn_3_O_4_ valence band obtained with XPS or using the MS method, and they were not studied with photoelectrochemical methods. In the first case, such an assessment leads to clearly overestimated values. Thus, the theoretically calculated value was about −2 V vs. E_H+/H2_ [15], −1.14 eV vs. NHE [17], −1.2 V vs. RHE [62]. Data obtained with the MS method show higher values and a large spread: −0.97 V vs. NHE (=−0.56 V vs. RHE) [13], −0.94 V vs. NHE (=−0.53 V vs. RHE) [32], −0.6 V vs. NHE (=−0.19 V vs. RHE) [33], −1.1 V vs. NHE (=−0.69 V vs. RHE) [16], −1.1 V vs. RHE [41], −0.23 V vs. NHE at pH 0 (=−0.23 V vs. RHE) [14]. Samples synthesized using citrate ions showed values close to ours: −0.3 V vs. SHE at pH 0 (=−0.3 V vs. RHE) [11].

E_OCP_(Et) values obtained with the addition of ethanol were found to be similar for all samples containing the Sn_3_O_4_ phase, which prompted us to plot these values in an energy diagram calculated from the data on the band gap and E_FB_ potentials (Figure 3d). Close E_OCP_ (dark yellow lines) values found for all samples (0.13–0.19 V) and a higher one (0.29 V) found for the TO4 sample having the SnO_2_ phase suggest that these potentials correspond to defect levels at which rapid recombination of the electron–hole pair occurs [60]. The presence of oxygen vacancies was reported for Sn_3_O_4_ at a potential of −0.3 V vs. NHE at pH 7 (=0.09 V vs. RHE) [33], which is close to the value found in our work. The E_OCP_(Et) (red lines) in the presence of ethanol do not reach the E_FB_, in contrast to the results obtained for this method in our previous work [60]. However, the values obtained for all samples containing the Sn_3_O_4_ phase are similar and coincide with the E_FB_ of the SnO_2_ (TO4) sample. We assume that interfacial electron transfer may occur from the E_FB_ levels from Sn_3_O_4_ in TO2 and TO3 samples to the E_FB_ level of SnO_2_. Unlike the OCP method, in the POP method, the electrode is polarized, and due to the excess of electrons, the influence of the recombination process is significantly reduced. For TO1, TO2 and TO4 samples, the effect of ethanol is not observed, and the E_POP_ potentials correspond to E_FB_. For the TO3 sample with a high content of the SnO_2_ phase, E_POP_ in a pure electrolyte is fixed at the level of E_FB_ SnO_2_ due to interfacial transfer and high recombination, and with the addition of ethanol, it coincides the E_FB_ level of Sn_3_O_4_ due to a decrease in recombination.

### 3.3. Photoelectrocatalytic Oxidation of Organic Substrates

**Figure 4:**
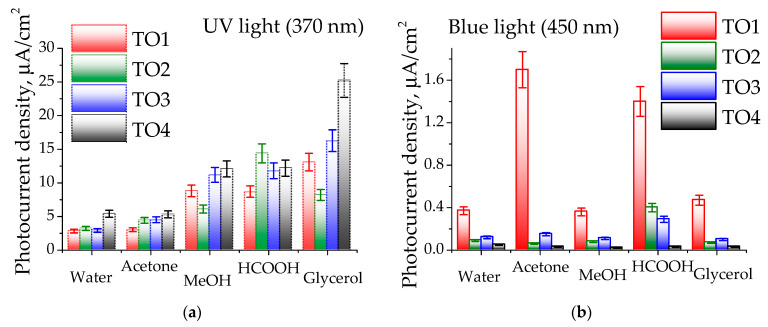
Photocurrents density in the electrooxidation of various organic substrates at a potential of 1 V RHE under (**a**) UV illumination (λ = 370 nm) and (**b**) visible light illumination (λ = 450 nm). Electrolyte—1 M Na_2_SO_4_ + 0.1 M organic substrate.

The samples were studied in the photoelectrochemical oxidation of small organic molecules (methanol, acetone, glycerol and formic acid). Experiments were carried out at potentials of 0.6, 1 and 1.4 V and at wavelengths of 370 and 450 nm. Dependencies of the current density on time at potential of 1 V vs. RHE are presented in Figure S4. The photocurrent values showed an insignificant increase with the potential (Figure S5). The exception is the TO2 sample, where the current in the oxidation reaction of formic acid under visible light irradiation sharply decreases with increasing potential, which may be due to the deactivation of the catalyst that resulted from the oxidation of Sn^2+^ ions. Under the influence of UV radiation, the catalysts operate stably and a slight increase in the photocurrent from the potential is observed. Photocurrents were compared at a potential of 1 V vs. RHE. The data are presented in Figure 4.

In general, the photocurrents of samples under UV illumination is several times higher than that found under visible light, which is associated with higher light absorption by the sample. For most materials, it increases in the series water = acetone < methanol < formic acid < glycerol. A comparison of the materials with each other shows that SnO_2_ (TO4) exhibits the greatest activity in all reactions, except for the oxidation of formic acid, which is explained by the highest potential of the TO4 valence band (Figure 3d). Under visible light irradiation, for all substrates (except formic acid), the activity correlates with the level of the valence band: TO1 (2.52 eV) > TO3 (2.42 eV) > TO2 (2.20 eV). The TO4 sample, representing SnO_2_, does not absorb visible light, so its photocurrent values are at the detection levels. The photocurrents of TO2 and TO3 samples in the oxidation under visible light of all substrates do not depend on the addition of an organic substrate and correspond to the photocurrents of water oxidation. TO1 is the most active one, and its photocurrent falls in the order acetone > HCOOH ≫ glycerol > methanol = water. The oxidation of formic acid probably proceeds with a different mechanism since all samples are active under both UV and visible light, and the series of photocurrent values differs depending on the type of illumination: TO2 > TO4 > TO3 > TO1 (UV irradiation) and TO1 > TO2 > TO3 (visible light).

## 4. Discussion

Additions of organic compounds as a sacrificial agent—electron donors—are widely used to increase the yield of hydrogen in photoreforming reactions [63]. The mechanism is the efficient trapping of photoinduced holes, which leads to a sharp decrease in recombination and an increase in the number of free electrons that participate in the formation of hydrogen from water [64]. The amount of hydrogen released during ultraviolet irradiation in an alcohol solution is determined by the number of hydrogen atoms located near the OH groups and decreases in the order glycerol > ethylene glycol > methanol > ethanol [63,65], which is consistent with the results obtained in our work for alcohol compounds (methanol and glycerol) under UV illumination. Oxidation of organic molecules on a surface occurs through a series of successive stages of electron transfer from the adsorbed molecules to the semiconductor. The ref. [66] presents the oxidation pathways of various molecules. Methanol is adsorbed to form methoxy groups, which are successively oxidized to formaldehyde and formate groups. Acetone is successively converted into acetate groups, then into formate groups. Formic acid is adsorbed as formate and oxidized to CO_2_ and H_2_O [66].

The low rate of oxidation of acetone under UV irradiation is due to the lower affinity for the surface adsorption of acetone than of alcohols [67,68]. The desorption of acetone from the surface of TiO_2_ as an intermediate product of the photooxidation of isopropanol was observed in work [68]. The high efficiency of HCOOH oxidation is due to the low isoelectric point of Sn_3_O_4_, which lies in the acidic region at pH = 2.5–3 [18] and the acidic nature of formic acid, which leads to strong adsorption of formate ions on the surface of the material, which are more reactive than molecular particles [69]. The resulting series of activity in the oxidation of HCOOH is most likely associated with the influence of the surface pH of the samples. Using formic acid as a sacrificial agent for hydrogen evolution under UV light on strontium titanates resulted in more hydrogen evolution than using methanol [70], which is consistent with our results.

**Figure 5:**
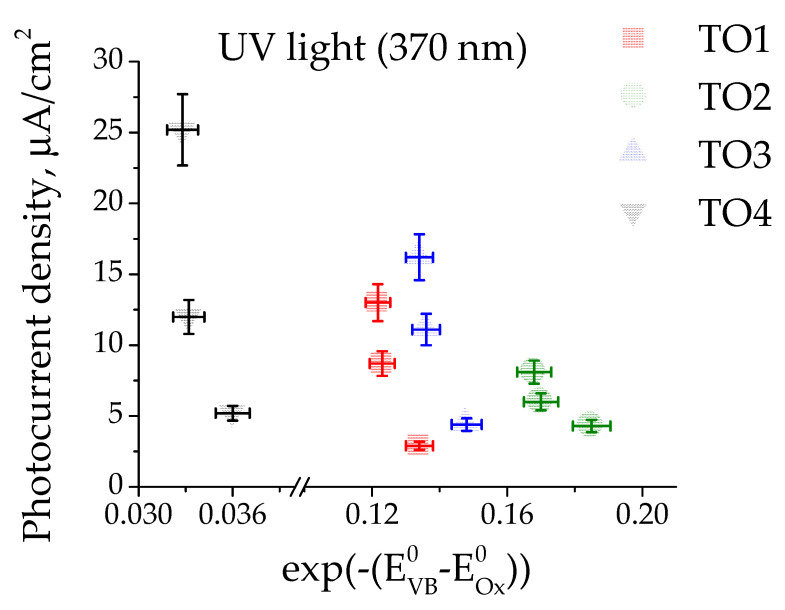
Dependence of photocurrents density in ultraviolet light regions on the difference in the oxidation potentials of organic substrates (E^0^_ox_) and the valence band of the samples (E_VB_). Symbols and colors indicate the following samples: red squares—TO1, olive circles—TO2, blue triangles—TO3, black inverted triangles—TO4. Photocurrents of formic acid are not shown here.

To clarify the influence of the valence band potential on the measured photocurrents, the oxidation potential of organic compounds was assessed, and the dependence was plotted in coordinates photocurrent-exp(−(E^0^_ox_−E_VB_)) (Figure 5), as proposed for alcohols in work [65]. A detailed description of potential calculations is provided in the Supplementary File and Table S2.

With the increase in difference between the valence band of the samples and the oxidation potential of organic compounds, an increase in photocurrents is observed, which is consistent with the hydrogen evolution increase reported in ref. [65]. For each substrate and each sample, the photocurrent under UV illumination increases with increasing valence band potential or decreasing substrate oxidation potential, respectively. The behaviour of acetone and formic acid are different. In the first case, low photocurrent values are associated with a low degree of acetone adsorption. In the second case, the surface acidic properties of the samples play a more decisive role in oxidation. Under visible light, a similar trend is observed.

## 5. Conclusions

In this work, a series of Sn_3_O_4_, SnO_2_ and Sn_3_O_4_/SnO_2_ samples with different phase ratios were prepared using hydrothermal synthesis. The structure of the samples was confirmed with XRD and Raman spectroscopy. The HRTEM method showed that SnO_2_ nanoparticles are in close contact with Sn_3_O_4_ nanosheets. DRS data showed that all samples containing the Sn_3_O_4_ phase absorb light in visible region. Flat-band potential measurements showed that the intermittent illumination (photocurrent onset potential) method with the addition of a sacrificial agent (ethanol) makes it possible to determine the E_FB_ for all samples, while the potential determined with the OCP method most likely corresponds to either the E_FB_ of SnO_2_ nanoparticles in the Sn_3_O_4_/SnO_2_ samples due to the possibility of interfacial electron transfer to tin dioxide or the potential of defective centers, where charge recombination occurs. The flat-band potentials for samples containing Sn_3_O_4_ were determined to be −0.43 V vs. RHE, while that for SnO_2_ was −0.15 V vs. RHE. The samples were studies in photo-electrocatalytic oxidation of a series of organic substrates (acetone, methanol, glycerol, formic acid) at a potential of 1 V vs. RHE under irradiation with ultraviolet (λ = 370 nm) and visible (λ = 450 nm) light. The presence of the SnO_2_ phase was shown to increase oxidation photocurrents in the ultraviolet region and to decrease those in the visible region. It was found that the photocurrent magnitudes can be related to the potentials of both the valence band of materials and oxidation of the substrate. For acetone, low photocurrent values were supposed to be associated with a low adsorption value. In the case of formic acid, the acidic properties of the surface are of decisive importance. Under visible light, the Sn_3_O_4_ sample showed high activities in the acetone and formic acid photo-electrooxidation, while the Sn_3_O_4_/SnO_2_ samples exhibited noticeable activity only in the oxidation of formic acid.

## Figures and Tables

**Table 1:** Surface chemical composition of the samples according to decomposition of XPS spectra.

Sample	Sn^2+^, % ^1^	Sn^4+^, % ^1^	O1, % ^2^	O2, % ^2^	Surface Composition
TO1	54	46	81	19	SnO_1.25_
TO2	38	62	94	6	SnO_1.3_
TO3	28	72	91	9	SnO_1.43_
TO4	0	100	81 ^3^	19 ^4^	SnO_1.88_

^1^ In relation to the Sn content, ^2^ In relation to the O content, ^3^ Lattice oxygen, ^4^ Defective oxygen.

**Table 2:** Results obtained with XRD, DRS and low-temperature nitrogen adsorption methods. The phase composition was estimated from the ratio of the integral intensities of the diffraction maxima (111) for Sn_3_O_4_ and (110) for SnO_2_.

Sample	CSR ^1^, nm		Phase Composition, %		Band-Gap Width, eV	S_BET_^2^, m^2^/g	V_pore_^3^, cm^3^/g
	Sn_3_O_4_	SnO_2_	Sn_3_O_4_	SnO_2_			
TO1	27	-	100	0	2.94	38.5	0.077
TO2	24	9	46	54	2.64	63	0.131
TO3	12	5.5	38	62	2.86	95.9	0.098
TO4	-	3	0	100	3.98	2.05	0.008

^1^ Coherent scattering region; ^2^ specific surface area determined by Brunauer–Emmet–Teller method; ^3^ specific pore volume.

**Table 3:** Values of potentials (V vs. RHE) measured by MS (E_MS_), POP (E_POP_) and OCP (E_OCP_) methods as well as E_FB_ values corresponding to E_POP_ obtained in ethanol-containing electrolyte E_POP_(Et).

Sample	E_MS_, V	E_POP_, V	E_OCP_, V	E_FB_, V
TO1	−0.06 ± 0.02	−0.38	0.127	−0.42
TO1 + EtOH	−0.04 ± 0.02	−0.42	−0.133	
TO2	−0.04 ± 0.04	−0.39	0.163	−0.44
TO2 + EtOH	−0.01 ± 0.03	−0.44	−0.155	
TO3	0.04 ± 0.03	−0.14	0.191	−0.44
TO3 + EtOH	0.01 ± 0.03	−0.44	−0.131	
TO4	−0.11 ± 0.03	−0.05	0.295	−0.15
TO4 + EtOH	−0.26 ± 0.04	−0.15	0.121	

## Data Availability

Data are contained within the article and supporting information.

## Supplementary Materials

The following supporting information can be downloaded at: https://www.mdpi.com/article/10.3390/ma16237300/s1, Figure S1: Decomposition of the obtained XPS bands in the Sn3d and O1s energy regions; Figure S2: Dependence of capacitance on potential in Mott–Schottky coordinates (MS method) in pure electrolyte and with ethanol additives; Figure S3. Intermittent irradiation (photocurrent onset potential, POP method) of samples in pure electrolyte and with the addition of ethanol; Figure S4. Dependence of current densities on the time at a potential of 1 V vs. RHE in the electrooxidation of water, acetone, methanol, formic acid and glycerol under irradiation with ultraviolet at 370 nm and visible at 450 nm light; Figure S5. Dependence of photocurrents on the potential in the electrooxidation of water, acetone, methanol, formic acid and glycerol under irradiation with ultraviolet at 370 nm and visible at 450 nm light. Table S1: Comparison of the literature’s data and experimental results of lattice parameters of the Sn_3_O_4_ phase in sample TO1 and the SnO_2_ phase in other samples; Table S2: Standard oxidation potentials (versus NHE) calculated for the different organic substrates; Table S3: The E_FB_, band-gap and E_CB_ values obtained in this work for TO1–TO4 samples.
