# Supplementary material for: Flat-Band Potential Determination and Catalytical Properties of Sn3O4/SnO2 Heterostructures in the Photo-Electrooxidation of Small Organic Molecules under Ultraviolet (370 nm) and Blue (450 nm) Light
Source: Materials (Basel). 2023 Nov 23;16(23):7300. doi: 10.3390/ma16237300 (PMC10707576; doi:10.3390/ma16237300)
Supplement: Supplementary file 1 [file materials-16-07300-s001.zip › materials-2700906-supplementary.pdf]

Supporting Information

Flat-band potential determination and catalytical properties of Sn_3O_4/SnO_2 heterostructures in the photo-electrooxidation of small organic molecules under acetone, methanol, glycerol and formic acid under Ultraviolet (370 nm) and Blue (450 nm) light.

Evgeny Gribov [1,*], Evgeny Koshevoy [1], Aleksey Kuznetsov [1], Maxim Mikhnenko [1], Evgeny Losev [1,2,3] and Mikhail Lyulyukin [1,2]

[1] [1]Boreskov Institute of Catalysis SB RAS, pr. Lavrentieva 5, Novosibirsk, Russia
[2] Novosibirsk State University, Pirogova str. 1, Novosibirsk, Russia
[3] Voevodsky Institute of Chemical Kinetics and Combustion SB RAS, , Institutskaya str. 3, Novosibirsk, Russia
* Correspondence: gribov@catalysis.ru; Tel.: +7 (383) 326-94-32

Table S1. Comparison of the literature's data and experimental results of lattice parameters of the Sn_3O_4 phase in sample TO1 and the SnO_2 phase in other samples.

**Phases**	**a, Å**	**b, Å**	**c, Å**	**α, °**	**β, °**	**γ, °**	**V, Å³**
PDF #16-0737 (Sn_3O_4)	5.88	8.2	4.86	93.35	91	93	234
TO1	5.90(2)	8.26(6)	4.89(2)	93.0(6)	90.1(5)	93.4(6)	238
PDF #41-1445 (SnO_2)	4.737		3.186				71.49
TO2	4.752(3)		3.183(5)				71.88
TO3	4.79(2)		3.15(3)				72.27
TO4	4.75(3)		3.18(4)				71.75

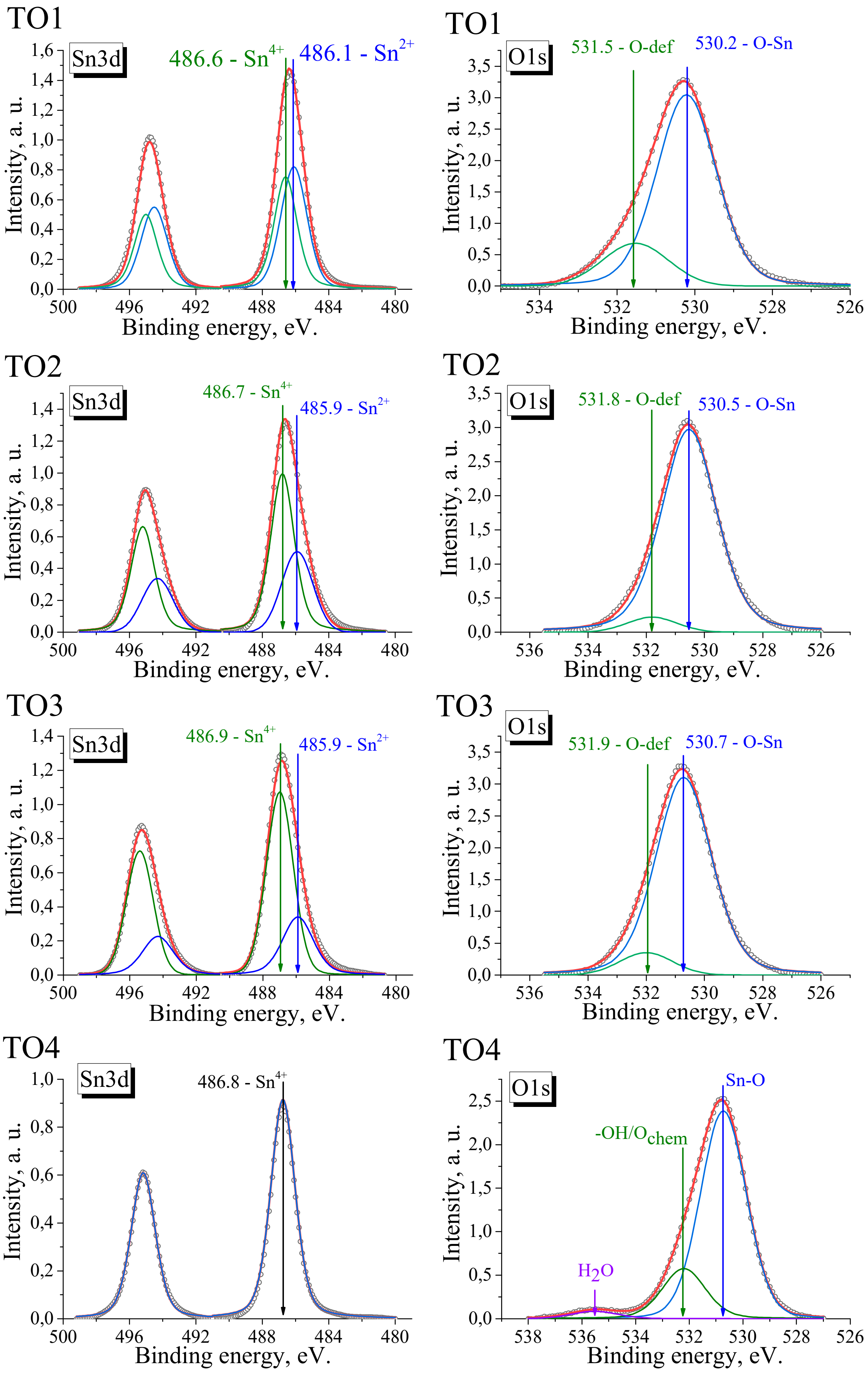

Figure S1. Decomposition of the obtained XPS bands in the Sn3d (left) and O1s (right) energy regions.

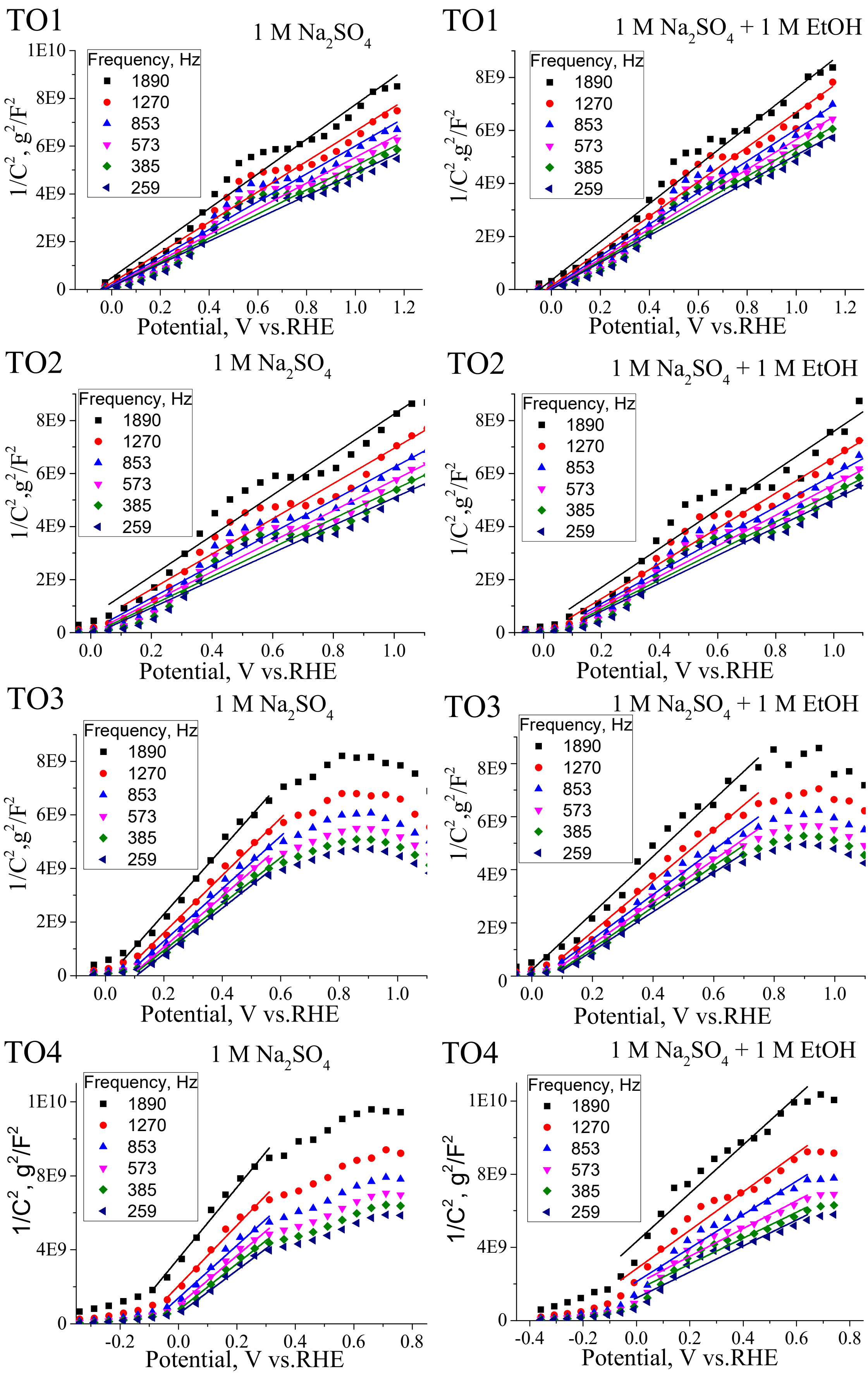

Figure S2. Dependence of capacitance on potential in Mott–Schottky coordinates (MS method) in pure electrolyte (left) and with ethanol additives (right).

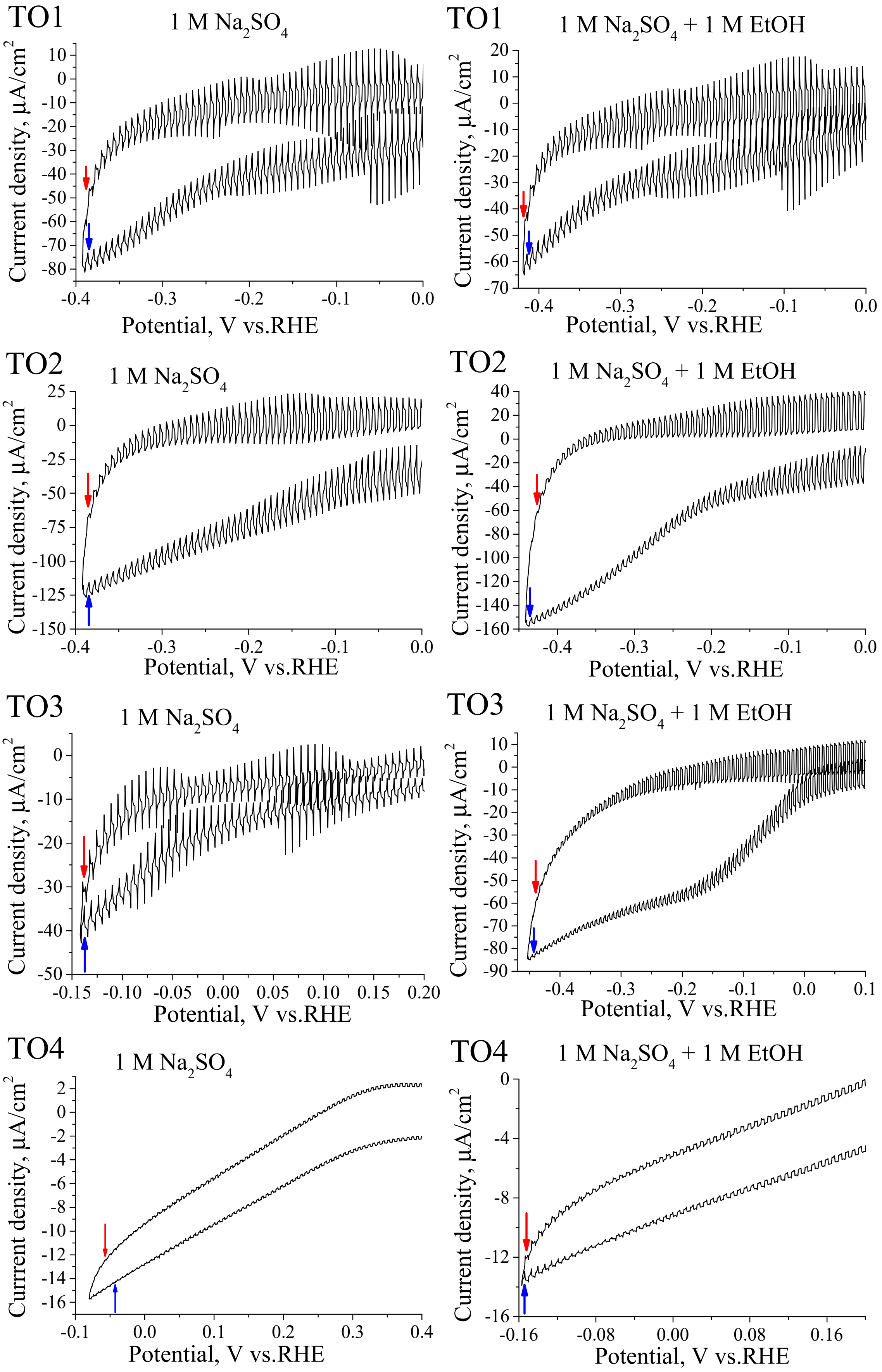

Figure S3. Intermittent irradiation (photocurrent onset potential, POP method) of samples in pure electrolyte (left) and with the addition of ethanol (right). Scan rate is 1 mV/s, 3 s—illumination, 3 s—dark phase, λ=370 nm. The arrows show the photocurrent onset potentials for the anodic (red) and cathodic (blue) scans.

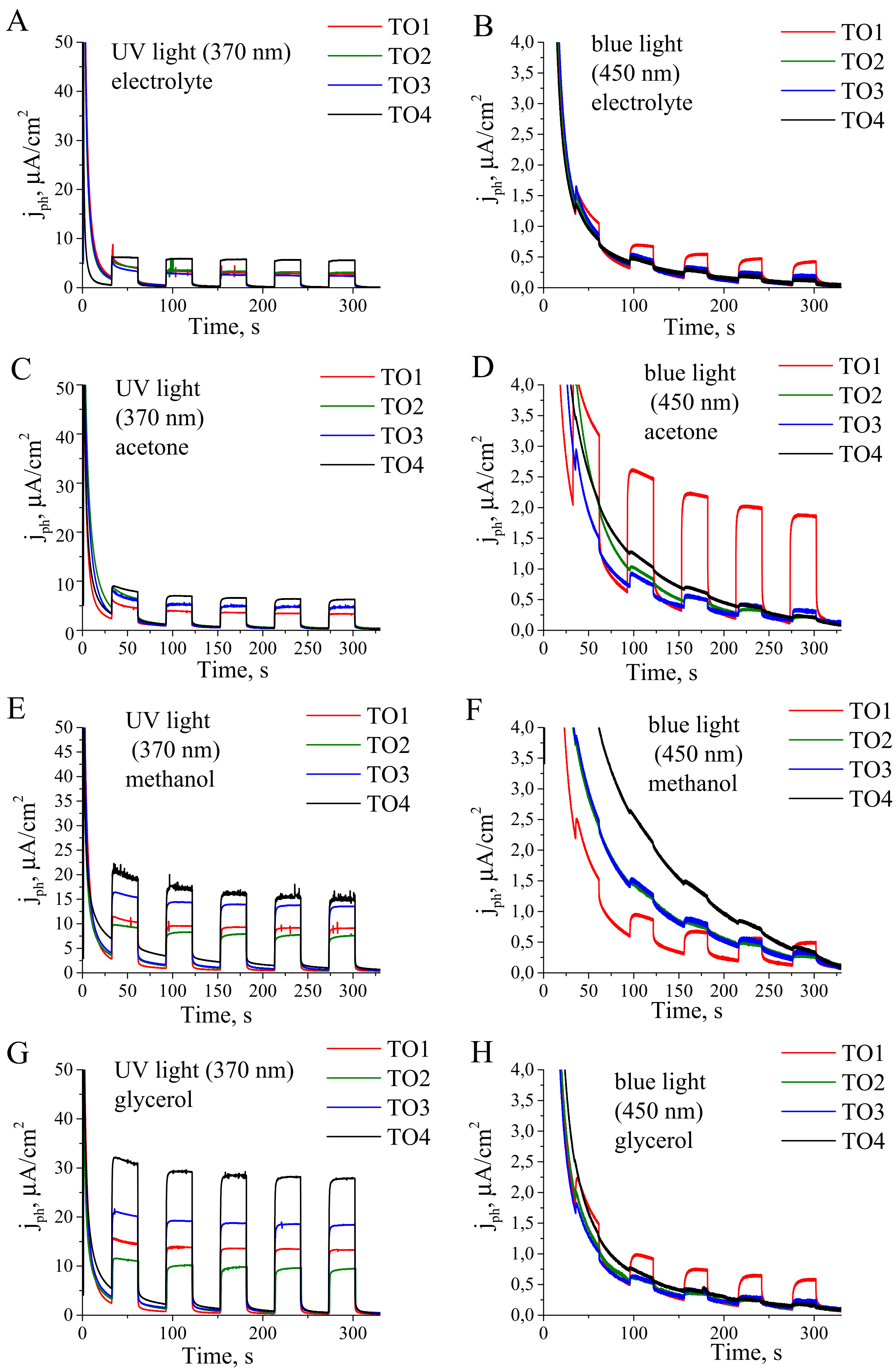

Figure S4. Dependence of current densities in the electrooxidation of water (A, B), acetone (C, D), methanol (E, F), glycerol (G, H) and formic acid (I, J) and on the time under irradiation with ultraviolet 370 nm (A, C,

E, G, I) and blue 450 nm (B, D, F, H, J) light. Potential is 1 V vs. RHE. Electrolyte is 1 M Na_2SO_4 + 0.1 M organic substrate. Light on for 30 s—light off for 30 s. Curves were shifted in such a way that the values of current density at 330 s are the same for all curves.

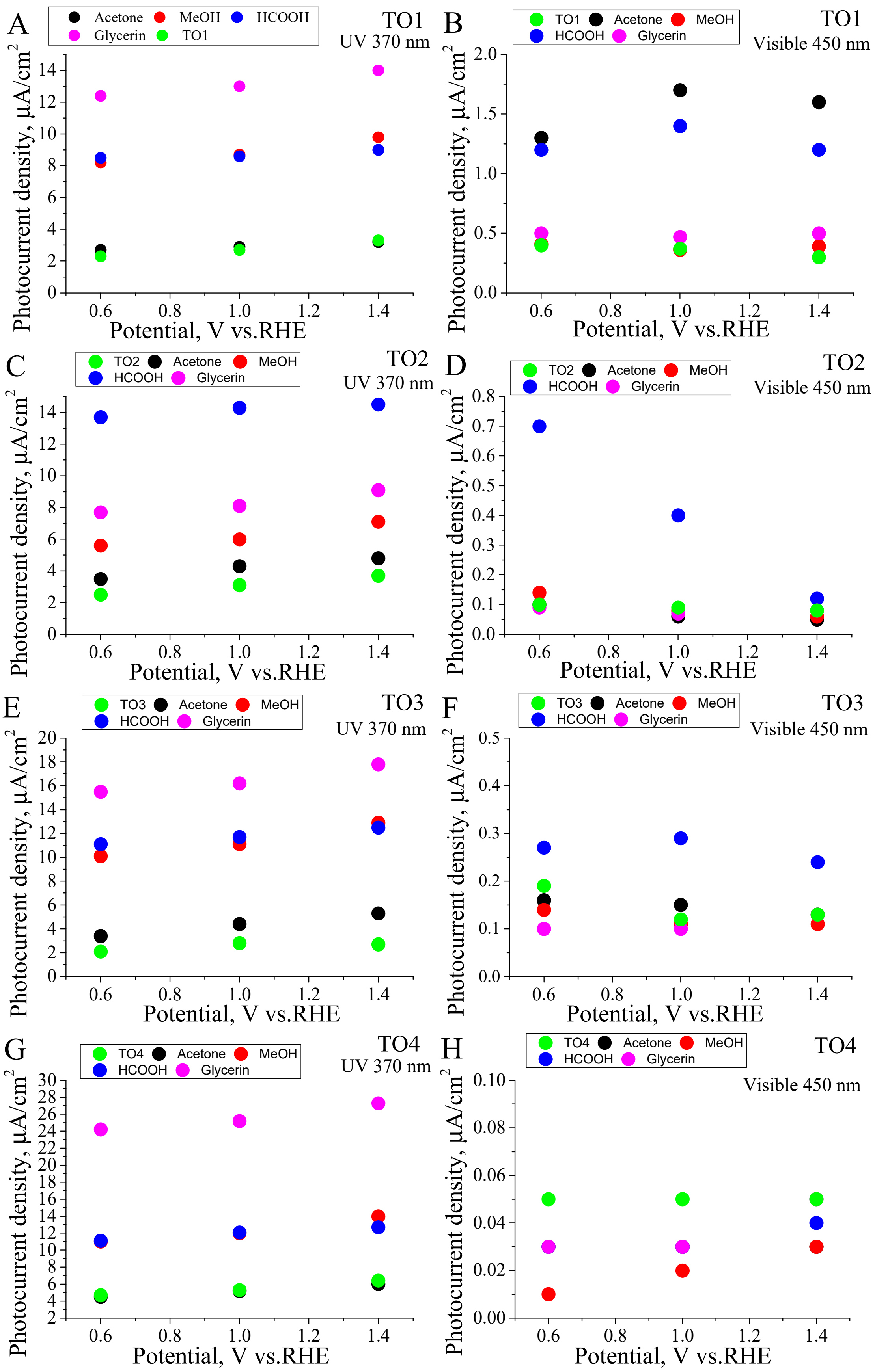

Figure S5. Dependence of photocurrents in the electrooxidation of water (green symbols), acetone (black symbols), methanol (red symbols), formic acid (blue symbols) and glycerol (magenta symbols) on the potential under irradiation with ultraviolet 370 nm (left) and visible 450 nm (right) light. Electrolyte is 1 M Na_2SO_4 + 0.1 M organic substrate. Light on for 30 s—light off for 30 s.

Estimation of oxidation potential of organic substrates

General reactions that we used for calculations:

$C_xH_yO_z + (2x\text{-}z)H_2O \rightarrow xCO_2 + nH^+ + ne^-$

Methanol: $CH_4O + H_2O \rightarrow CO_2 + 6H^+ + 6e^-$

Acetone: $C_3H_6O_1 + 5H_2O \rightarrow 3CO_2 + 16H^+ + 16e^-$

Glycerol: $C_3H_8O_3 + 3H_2O \rightarrow 3CO_2 + 14H^+ + 14e^-$

Formic acid: $C_1H_2O_2 \rightarrow CO_2 + 2H^+ + 2e^-$

Δ_fG^o values are taken from "Dean, J. A. (ed.) Lange's Handbook of Chemistry, 15th ed"
$-\Delta_rG^o = x\Delta_fG^o\,(CO_2) - \Delta_fG^o\,(C_xH_yO_z) - (2x\text{-}z)\,\Delta_fG^o\,(H_2O)$
E_{ox}^o (V) NHE = $-\Delta_rG^o/nF$, where n= (4x-2z+y)

$\Delta_fG^o\,(CO_2)$ = -394.4 kJ/mol; $\Delta_fG^o\,(H_2O)$ = -237.1 kJ/mol; F = 96485 C/mol

Table S2. Standard oxidation potentials (versus NHE) calculated for the different organic substrates

**Substrate**	**Δ_fG^0, kJ/mol**	**Δ_rG^0, kJ/mol**	**Eox^0, V NHE**
Acetone	-152.7	-155	0.100
Methanol	-166.6	-9.3	0.016
Formic Acid	-361.4	33	-0.171
Glycerol	-477	-5.1	0.004

The values of E_{VB} RHE obtained in this work for TO1–TO4 samples were recalculated to E_{VB} NHE as follows E_{VB} NHE = E_{VB} RHE + 0.413. The VB potentials were determined from our results as E_{VB} (V RHE) = E_{FB} (V RHE) + Band gap (eV). E_{FB} (V RHE) and band gap (eV) were taken from Table 4 and Table 3, respectively. Data are present in Table S2.

Table S3. The E_{FB}, band gap and E_{CB} values obtained in this work for TO1–TO4 samples.

Sample	E_{FB}, V vs. RHE	Band-gap, eV	EVB, V vs. RHE
TO1	-0.42	2.94	2.52
TO2	-0.44	2.64	2.2
TO3	-0.44	2.86	2.42
TO4	-0.15	3.98	3.83
